# Individualized rotation of left double lumen endobronchial tube to improve placement success rate: a randomized controlled trial

**DOI:** 10.1186/s12931-024-02799-x

**Published:** 2024-04-25

**Authors:** Huiying Zhou, Yuda Fei, Yuelun Zhang, Xiang Quan, Jie Yi

**Affiliations:** 1 Department of Anaesthesiology, Peking Union Medical College Hospital, Chinese Academy of Medical Sciences and Peking Union Medical College, No.1 Shuaifuyuan, Dongcheng District, Beijing, China; 2 Medical Research Centre, Peking Union Medical College Hospital, Chinese Academy of Medical Sciences and Peking Union Medical College, Beijing, China

**Keywords:** Airway management, Anatomy, Left double lumen tube, Rotation, Success rate

## Abstract

**Background:**

In conventional practice, the left double lumen tube (DLT) is rotated 90° counterclockwise when the endobronchial cuff passes glottis. Success rate upon the first attempt is < 80%, likely owing to varying morphology of the bronchial bifurcation.

**Methods:**

We conducted a randomized controlled trial to compare 90° counterclockwise rotation versus individualized degree of rotation in adult patients undergoing elective thoracic surgery using left DLT. The degree of rotation in the individualized group was based on the angle of the left main bronchi as measured on computed tomography (CT). The primary outcome was the first attempt left DLT placement success rate.

**Results:**

A total of 556 patients were enrolled: 276 in the control group and 280 in the individualized group. The average angle of the left main bronchi was 100.6±9.5° (range 72° to 119°). The first attempt left DLT placement success rate was 82.6% (228/276) in the control group versus 91.4% (256/280) in the individualized group (*P*=0.02, χ2 test). The rate of carina mucosal injury, as measured at 30 min after the start of surgery under fibreoptic bronchoscopy, was significantly lower in individualized group than control group (14.0% versus 19.6%, *P*=0.041). The individualized group also had lower rate of postoperative sore throat (29.4% versus 44.0%, *P*<0.001) and hoarseness (16.8% versus 24.7%, *P*＜0.05).

**Conclusions:**

Individualized rotation of left DLT based on the angle of the left main bronchi on preoperative CT increased first attempt success rate in adult patients undergoing elective thoracic surgery.

**Trial registration:**

The trial is registered at Chinese Clinical Trial Registry (ChiCTR2100053349; principal investigator Xiang Quan, date of registration November 19, 2021).

## Summary at a glance

The first attempt success rate of left DLT using the conventional 90° counterclockwise rotating is < 80%. Individualized rotation of left DLT based on the angle of left main bronchus increased the first attempt success rate to 91.4% in adult patients with thoracic surgery.

## Key Points

Question: Whether the first attempt success rate of individualized left double lumen tube (DLT) rotation based on the angle of the left main bronchi is higher than the traditional intubation method of 90° counterclockwise rotation?

Findings: The first attempt success rate was 91.4% in the individualized group, which the first placement success rate by the traditional left DLT intubation method was 82.6% in the control group.

Meaning: Individualized rotation of left DLT increased the first attempt success rate versus the conventional 90° counterclockwise rotation.

## Introduction

Since the left main bronchus is longer than the right main bronchi, most anesthesiologists prefer the left double lumen tube (DLT) for pulmonary isolation except in surgeries involving the left main bronchi [1–4]. However, placement left DLT is more challenging due to the smaller size and larger angle relative to the trachea [5–7]. 

Conventional approach when using a left DLT is to rotate the left DLT 90° counterclockwise when the endobronchial cuff passes the glottis [9]. Under this approach, the success rate upon the first placement attempt is approximately 80%, likely due to the varying angle of the left main bronchi on the median sagittal and coronal planes [5, 10–13]. In an imaging study of 50 adult men and 50 adult women, the mean true inclination angle of the left main bronchus was 108.4° [14]. 

We therefore hypothesized that 90° counterclockwise rotation is not ideal for all patients and conducted a randomized controlled trial to compare 90° counterclockwise rotation versus individualized degree of rotation based on preoperative computed tomography (CT) in adult patients undergoing elective thoracic surgery using left DLT. Results of the trial are reported below.

## Methods

This was a single-centre, parallel-group, randomized controlled trial. The trial protocol was approved by the Ethics Committee of Peking Union Medical College Hospital (ZS-2448) in accordance with the Declaration of Helsinki and is registered at Chinese Clinical Trial Registry (ChiCTR2100053349; principal investigator Xiang Quan, date of registration November 19, 2021). Written informed consent was obtained from all patients prior to enrolment. Adult patients scheduled for elective thoracic surgery using left DLT were randomized at a 1:1 ratio to undergo intubation using 90° counterclockwise rotation versus individualized degree of rotation based on the angle of the left main bronchi as measured on CT prior to surgery. Key exclusion criteria included: predicted difficult airway, e.g., Mallampati grade ≥ III, Cormack-Lehane grade ≥ III with video laryngoscopy; space-occupying lesions in the trachea, severe cervical spine deformity.

### Randomization, concealment and blinding

Randomization sequence was generated by a statistician not involved in this trial otherwise. Concealment was conducted using sealed opaque envelopes (opened immediately prior to intubation). Patients were blinded to group assignment. Outcome assessors were not blinded.

### Intervention

**Fig. 1:**
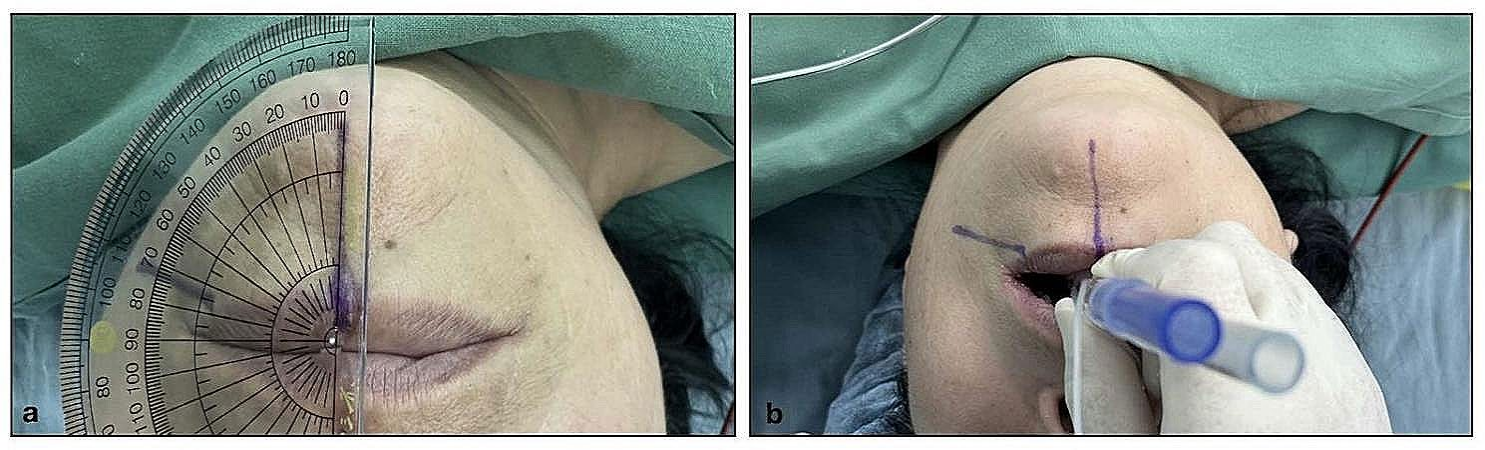
The method of left DLT rotation in one representative case. Measuring the true anatomic angle between the left main bronchus of a 62-year-old woman is 72°. 1a: Marking the left DLT rotation angle around the patient’s mouth. We defined 0° line is the line from the midpoint of the lip to the jaw; 1b: Rotating the left DLT according to the angle of lines

**Fig. 2:**
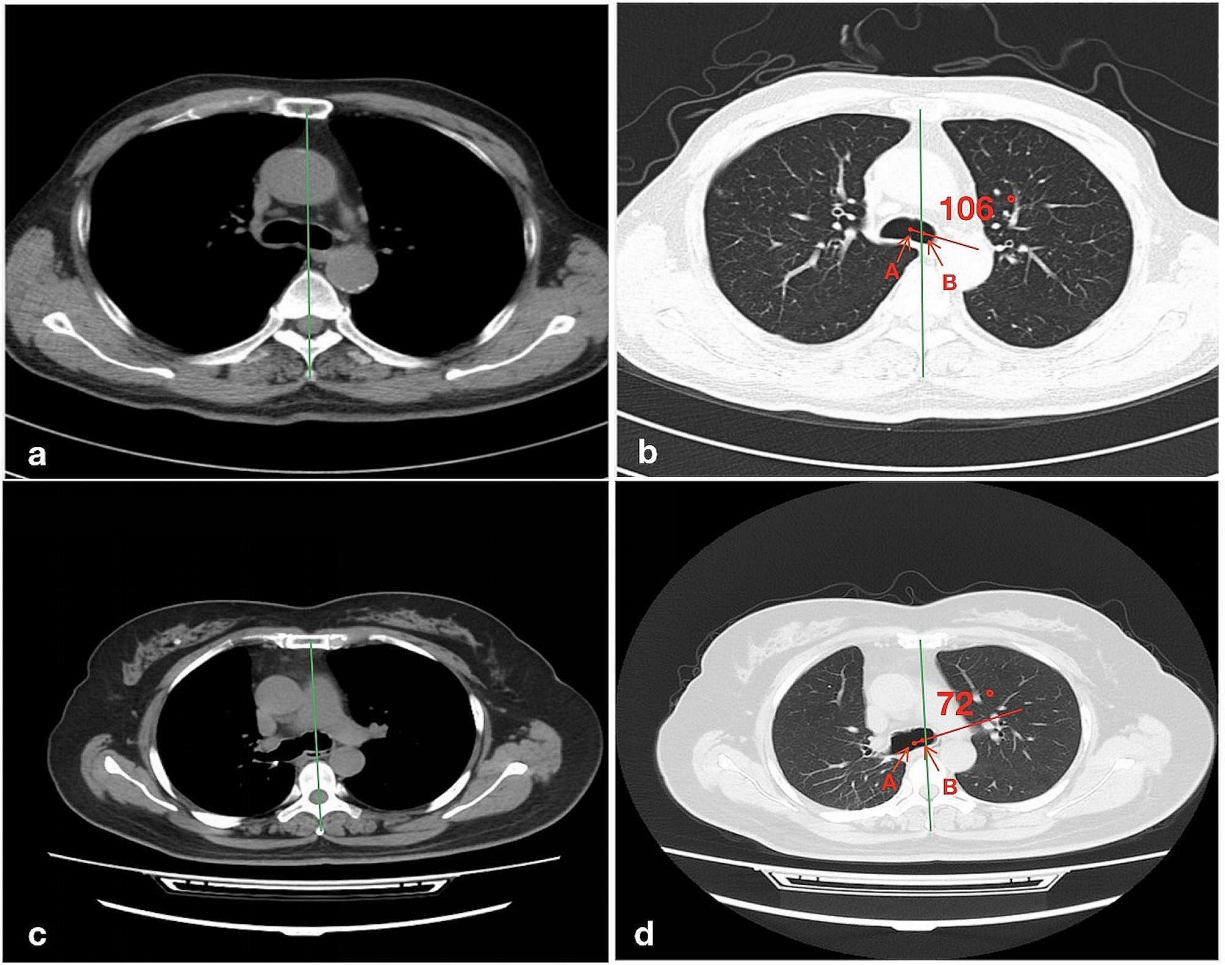
Determination of individualized degree for left DLT rotation in 2 representative cases. Panels a/b: a 74-year-old man; panels c/d: a 62-year-old woman. Green line: median sagittal reference line between sternum and vertebral body; red line was from the centre of the carina (**A**) to the centre of the left mainstem bronchi (**B**)

Intubation was conducted using a left DLT (Mallinckrodt™, Covidien LLC, MA, USA) by the attending anesthesiologists using video laryngoscopy (UE-TD-C, UE Medical Corp, Zhejiang, China). The rotation angle was marked around the patient’s mouth with a marker pen after grouping (Fig. 1a). When the endobronchial cuff of the DLT passed the glottis, the stylet was removed, and the DLT was rotated counterclockwise for 90° (for patients in the control group) or for the degree of rotation was based on the angle of the left main bronchi relative to the midline as measured on CT prior to surgery (in individualized rotation group). Then, the DLT was advanced until resistance was encountered. Lastly, the left DLT was connected to the circuit using the Y-connector. Degree of rotation in the individualized group was determined as explained in more details in Figs. 1 and 2. Fiberoptic bronchoscopy was used to verify the placement location of DLT in the left bronchi. Upon incorrect placement of the DLT, reintubation was conducted under fibreoptic bronchoscopy.

### Anesthesia

Surgery was conducted under general anesthesia using a routine protocol. Anesthesia was induced with 0.03 mg.kg^− 1^ midazolam, 2 µg.kg^− 1^ fentanyl, 2 mg.kg^− 1^ propofol, and 0.9 mg.kg^− 1^ rocuronium. Anesthesia was maintained with sevoflurane, fentanyl and rocuronium. The left DLT was removed when the operation was completed.

### Outcome measures

The primary outcome was success rate upon the first left DLT placement in the left bronchi. Secondary outcomes included the overall success rate of left DLT placement, carina mucosal injuries, sore throat and hoarseness.

The left DLT placement time was calculated from placement of the video laryngoscopy into mouth to left bronchus intubation until the DLT encountered resistance. Mucosal injury was assessed at 30 min after the start of surgery under fibreoptic bronchoscopy by an anesthesiologist unaware of the group assignment; sore throat and hoarseness was assessed at 24 h after surgery by a research staff unaware of the group assignment. Mucosal injury was graded into 3 levels: 1, redness as the red colour in the mucosa without surrounding swelling; 2, oedema as swollen mucosa; 3, hematoma as bleeding into mucosa [15, 16]. Severity of sore throat and hoarseness was graded using a numerical rating scale (NRS): 0, no sore throat; 10, the worst imaginable pain; and 0, no hoarseness; 10, the worst hoarseness [17]. 

### Statistical analysis

Assuming the success rates upon the first left DLT placement attempt in the control group and individualized group are 76% [5] and 86%, a sample size of 556 patients (278 per group) would provide 85.4% statistical power to detect a significant difference between groups with a 2-sided α of 0.05.

Categorical data are expressed as number (%) and analysed using χ^2^test. Continuous variables are expressed as mean ± standard deviation (SD) and analysed using Student’s t-test for independent sample. Two-sided *P* < 0.05 was considered statistically significant. All statistical analyses were conducted using SPSS (version 20.0; IBM, Chicago, IL, USA).

## Results

**Fig. 3:**
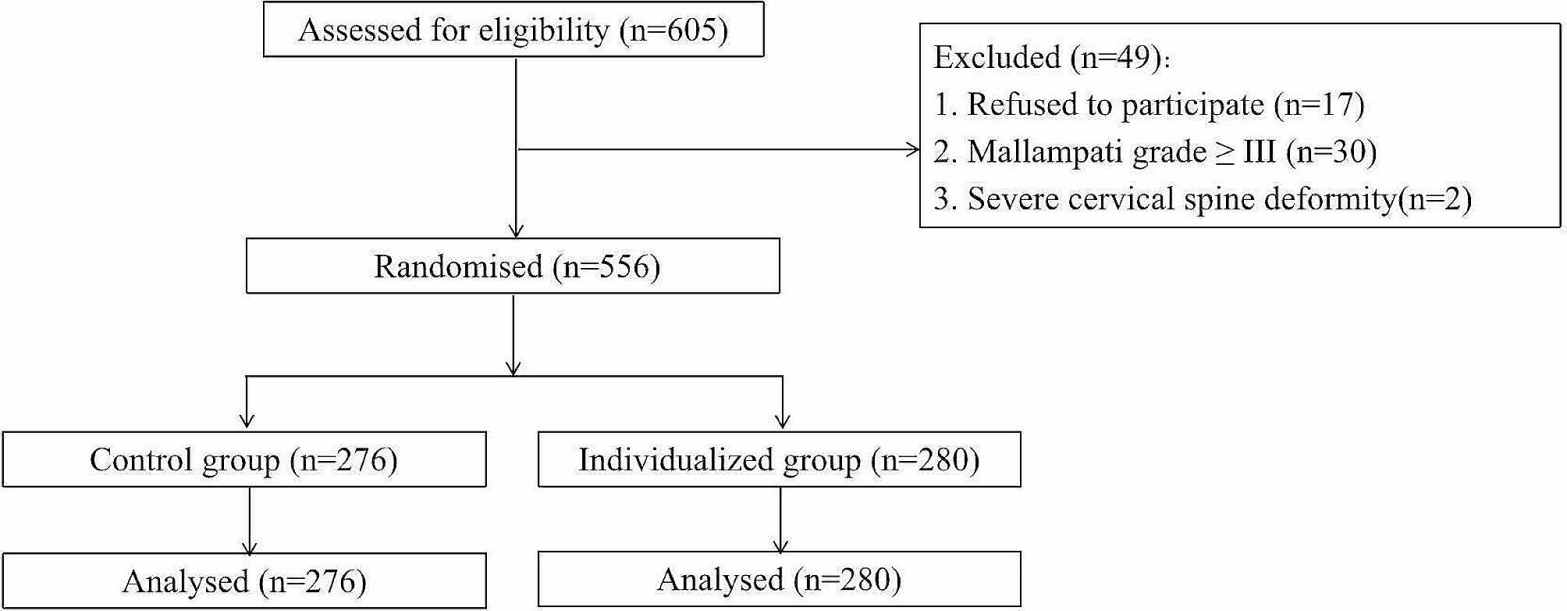
Patient flow through the trial

A total of 605 patients were assessed for eligibility during a period from December 2021 to June 2022 at Peking Union Medical College Hospital; 556 patients were enrolled: 276 in the control group and 280 in the individualized group (Fig. 3). The demographic and baseline characteristics are shown in Table 1.

**Table 1:** Demographic and baseline characteristics

Variables	Control group (*n* = 276)	Individualized group (*n* = 280)
Age (y)	57.0 ± 11.4	57.1 ± 11.1
Female sex, n (%)	143 (51.8%)	141 (50.4%)
BMI (kg/m^2^)	25.5 ± 3.6	25.0 ± 3.6
ASA physical status, n (%)		
I	30 (10.9%)	29 (10.4%)
II	199 (72.1%)	207 (73.9%)
III	47 (17.0%)	44 (15.7%)
DLT size, n (%)		
35 F	143 (51.8%)	141 (50.4%)
37 F	133 (48.2%)	139 (49.6%)
Mallampati grade, n (%)		
I	137 (49.6%)	142 (50.7%)
II	139 (50.4%)	138 (49.3%)
Cormack-Lehane grade, n (%)		
I	149 (54.0%)	150 (53.6%)
II	127 (46.0%)	130 (46.4%)
Type of surgery, n (%)		
Wedge resection	102 (40.0%)	116 (41.4%)
Segmentectomy	84 (30.4%)	83 (29.6%)
Lobectomy	90 (32.6%)	81 (29.0%)

Data are mean ± SD unless noted otherwise

Success rate upon the first left DLT placement was 82.6% (228/276) in the control group versus 91.4% (256/280) in the individualized group (relative risk: 1.107, 95% confidence interval: 1.039–1.186, *P* = 0.002, Table 2]. Overall success rate of left DLT placement was 100% in both groups. The first left DLT placement time did not differ between the control group (13.2 ± 6.1 s) and the individualized group(14.1 ± 8.5 s).

**Table 2:** The left DLT placement outcomes

	Control group (*n* = 276)	Individualized group (*n* = 280)	*P* value
First placement success, n (%)	228 (82.6%)	256 (91.4%)	0.002
First placement time (sec)	13.2 ± 6.1	14.1 ± 8.5	0.138
Duration of operation (min)	88.2 ± 36.7	92.7 ± 39.1	0.160
Duration of anesthesia (min)	110.9 ± 40.2	114.0 ± 42.3	0.400

Data are mean ± SD unless noted otherwise. Group comparison was conducted using χ^2^ test for first left DLT placement success rate, and Student’s t-test for all other variables

**Fig. 4:**
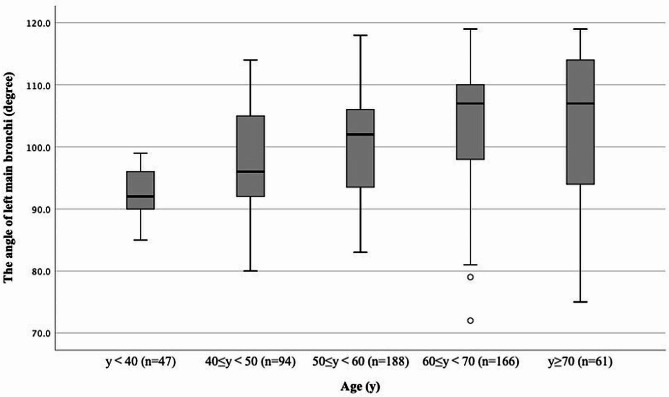
The angle of left main bronchus across age bracket. The box indicates interquartile range; the horizontal line within the box indicates group median; the whiskers extend to a distance of 1.5 times the interquartile range; the circles indicate outliers

The mean angle of the left main bronchus was 100.6 ± 9.5° in the overall cohort, 101.2 ± 9.3° in the control group and 99.7 ± 9.6° in the individualized group (*P* = 0.119). The mean angle was 101.4 ± 9.6° in women and 99.7 ± 9.3° in men (*P* = 0.031). The angle of the left main bronchus correlated positively with age: 92.4 ± 4.0° at < 40 years, 97.1 ± 8.1° at 40–50 years, 100.1 ± 8.2° at 50–60 years, 104.4 ± 9.3° at 60–70 years, 103.3 ± 12.2° at ≥ 70 years. Age specific angles are summarized in Fig. 4.

As the angle of left main bronchus correlated with increasing age, we conducted a subgroup analysis based on age. In the subgroup of ≥ 60 years, the first left DLT placement success rate was 77.2% (88/114) in the control group versus 89.4% (101/113) in the individualized group (relative risk: 1.158, 95% confidence interval: 1.031 to 1.317, *P* = 0.014, Table 3), while in the subgroup of < 60 years old, the first left DLT placement success rate was not statistically significant between the control group (86.4%, 140/162) and the individualized group(92.8%, 155/167) (Table 3).

**Table 3:** The left DLT placement first success rate outcomes across age

	Control group (*n* = 276)	Individualized group (*n* = 280)	*P* value
Patients ≥ 60 years old	88/114 (77.2%)	101/113 (89.4%)	0.014
Patients < 60 years old	140/162 (86.4%)	155/167 (92.8%)	0.057

Data are shown as number of success/total number (%). Group comparison was conducted using χ^2^ test for first left DLT placement success rate, and Student’s t-test for all other variables

Percentage of the patients with airway injury at the carina was 19.6% (54/276) in the control group versus 14.0% (39/280) in the individualized group (relative risk: 0.712, 95% confidence interval: 0.489–1.034, *P* = 0.041, Table 4). One patient in each group unexpectedly returned to the ICU after surgery and was therefore not available for assessment of sore throat and hoarseness at 24 h. The incidence of sore throat was 44.0% (121/275) in the control group versus 29.4% (82/279) in the individualized group (relative risk: 0.668, 95% confidence interval: 0.532–0.835, *P* < 0.001, Table 4). The incidence of hoarseness was 24.7% (68/275) in the control group versus 16.8% (47/279) in the individualized group (relative risk: 0.681, 95% confidence interval: 0.489–0.947, *P* = 0.011, Table 4).

**Table 4:** The left DLT placement complications

	Control group (*n* = 276)	Individualized group (*n* = 280)	*P* value
			*P* = 0.041
Mucosal injury, n (%)	54 (19.6%)	39 (14.0%)	
Grade 1	44 (16.0%)	29 (10.4%)	
Grade 2	10 (3.6%)	10 (3.6%)	
Grade 3	0 (0.0%)	0 (0.0%)	
	Control group (*n* = 275)	Individualized group (*n* = 279)	*P* value
Sore throat, n (%)	121 (44.0%)	82 (29.4%)	< 0.001
NRS severity	2.2 ± 0.9	2.3 ± 0.9	0.793
Hoarseness, n (%)	68 (24.7%)	47(16.8%)	0.011
NRS severity	1.7 ± 0.8	1.7 ± 0.8	0.909

NRS (numerical rating scale) severity. Data are shown as mean ± SD unless noted otherwise. Group comparison was conducted using χ^2^ test for the rate, and Student’s t-test for the severity

The patients with failed placement the left DLT at first attempt was older than the patients with successful placement at first attempt in the control group (60.7 ± 11.0 versus 56.1 ± 11.4 years, *P* = 0.011, Table 5), but not in the individualized group (57.0 ± 10.9 versus 58.0 ± 13.3 years, *P* = 0.675, Table 5).

**Table 5:** Comparison of the patients with left DLT first placement success and failure in two group

Variables	Control group (*n* = 276)		*P* value	Individualized group (*n* = 280)		*P* value
	Success (*n* = 228)	Failure (*n* = 48)		Success (*n* = 256)	Failure (*n* = 24)	
Age (y)	56.1 ± 11.4	60.7 ± 11.0	0.011	57.0 ± 10.9	58.0 ± 13.3	0.675
Female sex, n (%)	118(51.8%)	25(52.1%)	0.967	129(50.4%)	12(50.0%)	0.971
BMI (kg/m^2^)	25.5 ± 3.6	25.4 ± 3.5	0.862	25.1 ± 3.6	23.8 ± 3.5	0.105
DLT size, n (%)			0.967			0.971
35 F	118(51.8%)	25(52.1%)		129(50.4%)	12(50.0%)	
37 F	110(48.2%)	23(47.9%)		127(49.6%)	12(50.0%)	
Mallampati grade, n (%)			0.008			0.942
I	166(60.1%)	19(39.6%)		130(50.8%)	12(50.0%)	
II	110(39.9)	29(60.4%)		126(49.2%)	12(50.0%)	
Cormack-Lehane grade, n (%)			0.580			0.599
I	124(54.4%)	24(50%)		135(52.7%)	14(58.3%)	
II	104(45.6%)	24(50%)		121(47.3%)	10(41.7%)	

Data are mean ± SD unless noted otherwise.Group comparison was conducted using Student’s t-test and χ^2^ test

## Discussion

This trial demonstrated that individualized rotation of left DLT increased the success rate upon the first placement attempt in adult patients undergoing elective thoracic surgery using left DLT. Individualized rotation of left DLT also decreased the percentage of the patients with airway mucosal injury as assessed at 30 min after the start of the surgery, and the percentage of the patients with postoperative sore throat and hoarseness.

In a previous study of 1170 patients undergoing thoracic procedures under left DLT intubation with 90° counterclockwise rotation, success rate upon the first placement attempt was 75.9% [5]. The success rate upon the first placement attempt in the current trial was slightly higher at 82.7%. Such a discrepancy may likely reflects the use of video laryngoscope for intubation in the current trial [18–21].

The method of measuring the angle of the left main bronchus in the current trial differs from that in previous studies. Patel et al. measured the true anatomic angle between the left main bronchus and trachea; the angle was determined between the line that passes through the median sternum and median vertebral body at the level of the carina and the trajectory of the main bronchus based on three-dimension(3D) assisted CT imaging [14]. We used two-dimension(2D) CT images. Accordingly, the measured angle reflects the angle between median sagittal line and the left main bronchi rather than the true anatomical angle between the left main bronchus and the trachea. Increased success rate upon the first placement attempt in the individualized group versus the control group showed that, despite of the use of 2D CT only, the individualized degree of rotation is helpful. Also, the use of 2D rather than 3D CT images simplifies the procedure, and thus has potential for use in a wider setting.

The average angle of the left main bronchus has been reported to be 108.4° [14]. Consistent with the sex difference reported in a previous study, the angle of the left main bronchus was statistically higher in women (101.4 ± 9.6° versus 99.7 ± 9.3° in men) in the current trial. Also consistent with previous studies, the angle of the left main bronchus correlated with increasing age in the current study [10, 11, 22, 23]. Notably, the angle was 103.3 ± 12.2° in elderly patients (≥ 70 years of age) in contrast to 92.4 ± 4.0° in young patients (< 40 years of age). Moreover, highlighting the potential benefits on individualized DLT rotation in elderly patients. Although the left DLT size is an another factor for DLT placement [24, 25], our clinical trial was aimed to study the rotation angle of left DLT to improve the left DLT placement success rate at first attempt. Nevertheless, selecting appropriate DLT size based on CT measurement may further improve the efficacy of individualized DLT rotation.

The maximum angle of the left bronchus main bronchus in this trial was 119°, whereas the minimum angle was only 72°. In our opinion, such a broad range is likely one of the reasons for the lower rate of left DLT intubation success upon first attempt in the control group, and highlights the benefit of using an individualized degree of rotation. It took only 1 min to measure the rotation angle according to the angle between the median sagittal line and the left main bronchi in 2D CT in PACS (picture archiving and communication system). According to the subgroup analysis in elderly patients (≥ 60 years of age), we could adopt the individualized rotation of left DLT in the patients ≥ 60 years old to improve clinical efficiency and reduce clinical workload.

The use of left DLT is associated with increased damage to the airway [8, 26]. The rate of laryngeal and airway injury when using single lumen tube intubation has been reported to be 4–7%.^27^, ^28^ DLT in general is associated with higher rate of airway injury [8]. For left DLT using 90° counterclockwise rotation during intubation and placement, the rate of bronchial injury ranges from 25–35% [15, 16, 29]. The rate of airway injury in the control group in the current trial was slightly lower at 19.6%, likely due to the use of video laryngoscopy [19]. Consistent with the higher success rate upon the first placement attempt, the use of individualized degree of rotation resulted in a statistically significant reduction of airway injury by 5.6%. In comparison to the control group, the individualized group in this trial also had lower rate of sore throat and hoarseness. In addition to higher first left DLT placement success rate, lower degree of injury in the individualized group may also be attributed to better alignment between the left DLT and the left main bronchus.

This trial has several limitations. First, this was a single-centre trial. Whether the results could be generalized to a wider setting is unknown. Second, determination of an individualized angle is time-consuming despite of the use of 2D CT and places extra burden to patient management. It is likely that using a uniform degree of rotation higher than 90° based on the population mean is sufficient. Based on the results of the current trial and previous anatomical studies [14], we are now planning a trial using 100°-110° [14].

In conclusion, individualized rotation of left DLT based on the angle of the left main bronchus on preoperative 2D CT increased success rate upon the first placement attempt in adult patients undergoing elective thoracic surgery using left DLT.

## Data Availability

Data is provided within the manuscript.
